# Mycorrhizae and Rhizobacteria on Precambrian Rocky Gold Mine Tailings: I. Mine-Adapted Symbionts Promote White Spruce Health and Growth

**DOI:** 10.3389/fpls.2018.01267

**Published:** 2018-09-03

**Authors:** Martin B. Nadeau, Joan Laur, Damase P. Khasa

**Affiliations:** ^1^Viridis Terra Innovations Inc., Sainte-Marie, QC, Canada; ^2^Institut de Recherche en Biologie Végétale, Université de Montréal, Montreal, QC, Canada; ^3^Centre for Forest Research and Institute of Integrative and Systems Biology, Université Laval, Quebec City, QC, Canada

**Keywords:** mycorrhizae, mine waste, *Picea glauca*, rhizobacteria, plant growth, plant health

## Abstract

White spruce [*Picea glauca* (Moench) Voss] is a commercially valuable boreal tree that has been known for its ability to colonize deglaciated rock tailings. Over the last decade, there has been an increasing interest in using this species for the revegetation and successful restoration of abandoned mine spoils. Herein, we conducted a glasshouse experiment to screen mycorrhizal fungi and rhizobacteria capable of improving the health and growth of white spruce seedlings growing directly on waste rocks (WRs) or fine tailings (FTs) from the Sigma-Lamaque gold mine located in the Canadian Abitibi region. After 32 weeks, measurements of health, growth, and mycorrhizal colonization variables of seedlings were performed. Overall, symbionts isolated from roots of healthy white spruce seedlings growing on the mining site, especially *Cadophora finlandia* Cad. fin. MBN0213 GenBank No. KC840625 and *Pseudomonas putida* MBN0213 GenBank No. AY391278, were more efficient in enhancing seedling health and growth than allochthonous species and constitute promising microbial symbionts. In general, mycorrhizae promoted plant health and belowground development, while rhizobacteria enhanced aboveground plant biomass. The observed beneficial effects were substrate-, strain-, and/or strains combination-specific. Therefore, preliminary experiments in control conditions such as the one described here can be part of an efficient and integrated strategy to select ecologically well-adapted symbiotic microorganisms, critical for the success of a long-term revegetation program.

## Introduction

Mining operations generate an enormous volume of waste materials that are difficult to dispose of. With more than 200 active sites, the Canadian mineral extraction industry produces over 1,000 million tons of solid waste per year (Statistics Canada, 2012; Mining Association of Canada, 2016). Prior to the first legislation in the 1970s, proper mine closure plans were not required, and residues were usually stored on adjacent wasteland where they constitute a very challenging substrate for the regeneration of natural ecosystems.

In Val-d’Or, Québec, the Sigma-Lamaque gold mine has been in operation since 1935. No mine closure plan was in place, coarse WRs and FTs cover 150 ha within the city limits that must now be efficiently revegetated. To do so, the revegetation of this area considered at low risk for contamination (Beauregard et al., 2012; Callender, 2014; Nadeau et al., 2016) with native species can be a successful strategy (Jackson et al., 1995; Larchevêque et al., 2013; Nadeau et al., 2016). White spruce is a dominant species of the boreal forest. Due to its ability to repopulate harsh environments and promote the subsequent establishment of a self-sustaining and more diverse ecosystem (Sutton, 1973), it is commonly used for land reclamation (Renault et al., 2004; Leewis et al., 2013; Onwuchekwa et al., 2014; Schoenmuth et al., 2015; Frerichs et al., 2017). A few healthy white spruce [*Picea glauca* (Moench) Voss] seedlings found naturally regenerating on the mine tailings revealed a mycorrhizal fungal community distinct from the neighboring ecosystems (Nadeau et al., 2016). Beneficial microorganisms discovered from the rhizosphere of seedlings can significantly ease plant growth and development – a major asset in a nutrient-depleted substrate like the Sigma-Lamaque gold mine tailings (Nguyen et al., 2006; Hoeksema et al., 2010). For instance, in tailings of a copper mine, fungal inoculation enhanced Japanese red pine (*Pinus densiflora*) seedlings performance (Zong et al., 2015). Similarly, in western Canada, Onwuchekwa et al. (2014) have shown that the inoculation of white spruce and jack pine (*Pinus banksiana*) with several fungal species (*Hebeloma crustuliniforme*, *Suillus tomentosus*, *Laccaria bicolor*) improved plant survival on oil sand tailings.

In addition to symbiotic fungi, rhizobacteria were also isolated from the rhizosphere of white spruce host naturally regenerating on the Sigma-Lamaque mining site. Like mycorrhizae, bacterial strains can increase plant performance as observed in coniferous tree species (Cardoso et al., 2011). As a matter of fact, the use of biofertilizers in agriculture is gaining popularity worldwide (Humphry et al., 2007; Baset Mia and Shamsuddin, 2010; Damir et al., 2011; Hrynkiewicz and Baum, 2011).

In nature, positive interactions between plant host and its symbionts occur through a number of mechanisms. Whether a beneficial microorganism is a biocontrol agent (Kropp and Langois, 1990; Pieterse et al., 2003), improves root development, water, and nutrient uptake (Blum et al., 2002; Allen, 2007; Vayssières et al., 2015) and/or limits the uptake of toxic compounds (Chaudhry et al., 2005), it co-exists with other organisms within the microbiome. Combinations of microbial strains or species may be neutral or even profitable to the plant host. However, some fungal and bacterial species can also behave like antagonists (Artursson et al., 2006; Uroz et al., 2007; Uroz et al., 2009). Plant–microbe interactions evolve with the development of a more complex ecosystem, with soil weathering and aging of the plant host (Mummey et al., 2002; Allen E.B. et al., 2003; Allen M.F. et al., 2003; Elliott et al., 2007).

White spruce has a substantial potential to be used in the phytorestoration of mine tailings. Because it is highly sensitive to transplanting shock (Nienstaedt and Zasada, 1990), the selection of adequate symbionts to improve the establishment of young seedlings could determine the success of a revegetation program with this species. Moreover, the role of mycorrhizal fungi and rhizobacteria in tree physiology on Precambrian metamorphic rocks of the Canadian Shield has never been studied. In the context of evaluating a new selection strategy for site-specific reforestation, we investigated the potential of selected cultivable fungi and rhizobacteria to improve the performance of white spruce seedling on mine tailings under glasshouse conditions. Thus, two hypotheses were formulated. First, the combined inoculation of seedlings with fungi and rhizobacteria improves the growth and overall health of seedling. Second, the use of native strains isolated directly from the mining site may give better results than the allochthonous ones.

## Materials and Methods

### Seed Germination and Seedling Growth

White spruce seeds were germinated in Styroblock containers. Cavities (9.5 mL capacity) were filled with a peat–vermiculite–perlite substrate (80:15:5). The trial was conducted in a greenhouse at the Université Laval (Quebec City, QC, Canada). The greenhouse was disinfected with a bleach solution prior to the experiment. To favor seedling establishment and nutrition before inoculation, plants were fertilized 2 weeks after germination with a commercial solution (20N-8P-20K). Three weeks after germination, seedlings were transferred into 1.75 L pots filled with WRs or FTs collected from Sigma-Lamaque gold mine (Val-d’Or, QC, Canada). The mine residues are considered to have low risk of contamination (Taner et al., 1986; Beauregard et al., 2012) but soil chemical composition analyses of four randomly selected samples indicate an absence of nitrogen source (${\text{NO}}_{3}^{-}$, ${\text{NO}}_{2}^{-}$, or ${\text{NH}}_{4}^{+}$), low concentration of elements important for plant growth (P: 0.203 ± 0.123(SE) g/kg; K: 0.096 ± 0.003 g/kg) and relatively important concentration of metals (Fe: 14.5 ± 0.5 g/kg; Ca: 22.3 ± 0.5 g/kg; Mg: 4.2 ± 0.2 g/kg; and Al: 5.7 ± 0.1 g/kg); arsenic (8.75 ± 0.25 mg/kg); and cyanides (4.6 ± 0.6 mg/kg). The pH of tailings was relatively alkaline with values varying between 8.55 and 8.68. Throughout the 32-week-long experiment, seedlings were watered daily at field capacity. Greenhouse conditions for optimal growth of white spruce seedlings were set at an alternating temperature of 25/20°C (day/night). Seedlings received artificial light with light intensity of 400 lux (5.56 μE m^−2^ s^−1^) for 16 h/day.

### Bacterial and Fungal Inoculation

Three bacterial strains were selected for this experiment. One commercial strain of *Azotobacter chroococcum* ATCC 9043 was purchased from CEDARLANE Laboratories, Ltd. (Burlington, ON, Canada). Two (*Pseudomonas putida* MBN0213 GenBank No. AY391278 and *Rhizobium radiobacter* MBN0213 GenBank No. FR828334) were isolated from the rhizosphere of healthy white spruce host naturally regenerating on coarse WRs of the mining site following the method described by Mazinani et al. (2013), a combination of the soil paste and the direct sowing of single soil grains on Mannitol-agar medium selective isolation methods for nitrogen-fixing bacteria. Bacteria were not screen for metal resistance. For accurate identification, DNA extraction, PCR amplification using standard 16S rRNA primers 27f and 1492r (Peace et al., 1994), and DNA sequencing were performed following the method employed by Herter et al. (2011).

For maximum cell production before inoculation, bacteria were cultivated in suspension cultures under aseptic conditions at 30°C for 7 days. *P*. *putida*, *R*. *radiobacter*, and *A*. *chroococcum* were respectively grown in Tryptic soybean broth – Difco medium, a liquid yeast extract mannitol medium and a liquid Waksman medium following the method developed by Agri-Tech (Aurangabad-Maharashtra, India). Bacterial cells were harvested after centrifugation (20 min, 4000 rpm at 4°C) and resuspended in sterile water until the inoculant reached a concentration of 10^8^ CFU mL^−1^. Ten milliliter of the inoculant was applied onto roots of 4-week-old white spruce seedlings two times within 14 days in order to increase rhizospheric colonization success.

Three mycorrhizal fungi displaying compelling *in vitro* growth and tolerance to mine tailings were chosen for this experiment (Nadeau, 2014). *Hebeloma crustuliniforme* UAMH5247, from the Centre for Forest Research genomic and microbial collections^1^, was isolated from white spruce roots in a natural forest stand of the boreal forest in Canada. Both *Tricholoma scalpturatum* Tri. scalp. MBN0213 GenBank No. KC840613 and *Cadophora finlandia* Cad. fin. MBN0213 GenBank No. KC840625 were isolated from healthy naturally regenerating white spruce seedlings on Sigma-Lamaque gold mine coarse tailings (Nadeau et al., 2016).

The inoculum was produced by cultivating fungal mycelia in a liquid Melin Norkrans medium at 23°C under aseptic shaking conditions. After 2 months, the mycelia were collected and rinsed with sterile water to discard excess nutrients. Blended mycelia were mixed with sterile water (ratio 1:10) to obtain a final concentration ≥5 × 10^5^ viable propagules mL^−1^. White spruce seedlings were inoculated with 5 ml of the inoculant when they were 3-week-old and a second time 4 weeks later to increase root inoculation success. The inoculum was released into the root zone using an analog adjustable-dispenser.

### Experimental Design and Treatments

The experimental design was a randomized complete block (RCB) with three crossed fixed factors (*Tailing type* × *fungi* × *bacteria*). Tailing type was composed of two levels: WR and FT. Fungal factor had a total of four levels: none (noF), *H*. *crustuliniforme* (*Hc*), *T*. *scalpturatum* (*Ts*), and *C*. *finlandia* (*Cf*). Bacteria also had four levels: none (noB), *P*. *putida* (*Pp*), *R*. *radiobacter* (*Rr*), and *A*. *chroococcum* (*Ac*). There were 32 treatments with three replicates in each of the four blocks for a total of 384 experimental units. Each replicate was randomly assigned to experimental units within blocks. Every experimental unit consisted of a 1.75-L pot filled with tailings containing one white spruce seedling. Experimental units within blocks were separated by a thin piece of plastic to avoid cross bacterial contamination. Each block was surrounded by two guard rows to maintain the most homogeneous environmental conditions possible in all experimental units. **Supplementary Figure S1** gives detailed layout and illustrations of the experimental design.

### Measurements of Seedling Survival Rate and Nutrient Content Analyses

Detailed descriptions of seedling survival rate and nutrient content analyses are presented in the companion paper (Nadeau et al., 2018).

### Measurements of Seedling Health and Growth

At the end of the glasshouse experiment, seedlings were brought into a growth chamber an hour before measuring chlorophyll fluorescence. Photochemical efficiency (Fv/Fm) was measured in a dark environment using a portable fluorometer PAM-2000 with the data acquisition software DA-2000 (Heinz Walz, Effeltrich, Germany). Briefly, the foliage was placed under the fluorescence booster for recording Fv/Fm data.

Needles were excised from stems, weighted, and individually positioned on a transparent plastic plate prior to scanning (WinSEEDLE PRO LA2400 scanner system and software, Regent Instruments, Inc., Quebec City, QC, Canada) were used to determine specific surface foliar areas (SSFA) of green, yellow, brown, dark-red, and light-red foliar tissues. Percentages of healthy-green foliage and dark-red foliage were calculated by comparing their SSFA with the sum of all SSFAs.

Stems were weighted and measured with a 15-cm ruler. Roots were washed gently with tap water in a 2-mm mesh sieve to remove all soil particles and thereafter weighed. The percentage of fungal colonization was calculated after manual counting under a microscope as the ratio of mycorrhizal root tips number to total root tips number multiplied by 100. Subsequently, roots were transferred onto a transparent plastic plate. WinRHIZO PRO LA2400 scanner system and software (Regent Instruments, Inc., Quebec City, QC, Canada) were used for measuring total root length, volume, and number of root tips.

For dry biomass analyses, white spruce seedling roots, shoots, and needles were dried at 65°C for 7 days. Percentage of water content was calculated by subtracting dry biomass from wet biomass, dividing the result by wet biomass and then multiplying by 100.

### Statistical Analyses

#### Differences Among Treatments

All the statistical analyses were conducted with the SAS software (SAS Institute Inc., 2012). Seedling health, growth, and percentage of fungal root colonization data were subjected to three-way analyses of variance (*Tailing type* × *fungi* × *bacteria*) using PROC GLM. Proper transformations were performed when needed. Log transformations were performed with total root length, number of root tips and dry biomass data. Arcsine transformation was used with the photochemical efficiency variable. Finally, non-parametric analyses (Wilcoxon rank sum test and *post hoc* test) was conducted on the percentage of dark-red foliage and percentage of roots colonized by fungus because it was not possible to meet normality and/or homoscedasticity assumptions even after transformations. The non-parametric tests were performed using PROC NPAR1WAY. Significance for all analyses was set at α = 0.05 (*P* ≤ 0.05). Means and standard errors of each treatment were calculated for all health, growth, and colonization variables.

#### Correlation Analyses

Correlations between the percentage of colonized roots and other health and growth variables were investigated using PROC CORR. Furthermore, correlation analyses between health variables (photochemical efficiency, percentage of healthy green foliage, and percentage of dark-red foliage) and growth variables (root, stem, and needle dry biomass) were performed in order to determine if there was a relationship between white spruce seedling health and growth. For these analyses, individual data were used. Significance for all Pearson correlation coefficients (*r*) was set at α = 0.05 (*P* ≤ 0.05).

## Results

### Effect of Symbiotic Association on Plant Health

**FIGURE 1:**
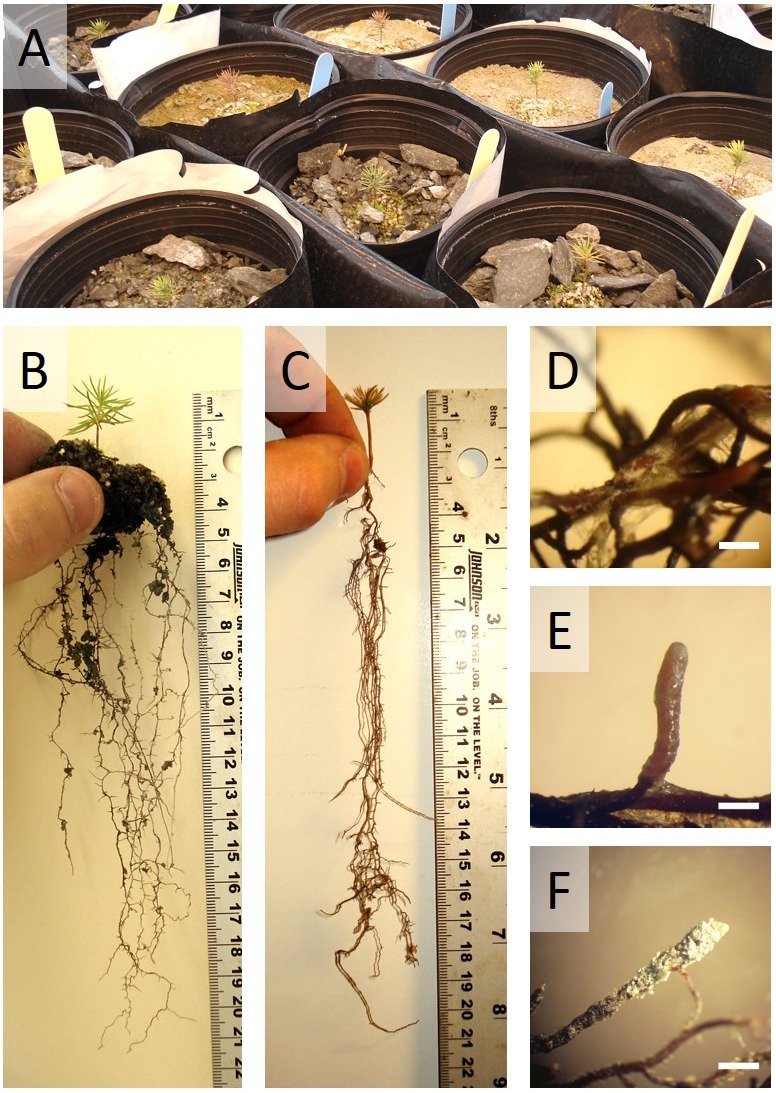
White spruce seedling after 32 weeks of growth in waste rocks or fine tailings from the Sigma-Lamaque gold mine. **(A)** Partial view of the glasshouse experiment; typical examples of **(B)** a healthy white spruce seedling and **(C)** an unhealthy white spruce seedling; root tips colonized by **(D)** *Hebeloma crustuliniforme*, **(E)** *Tricholoma scalpturatum*, and **(F)** *Cadophora finlandia*.

After the 32 weeks of glasshouse trial in Sigma-Lamaque gold mine tailings, seedling exhibited contrasting phenotypes (**Figure 1A**). Seedling growth and health clearly benefitted (**Figures 1B,C**) from symbiotic associations that prove to be successful (**Figures 1D–F**).

**FIGURE 2:**
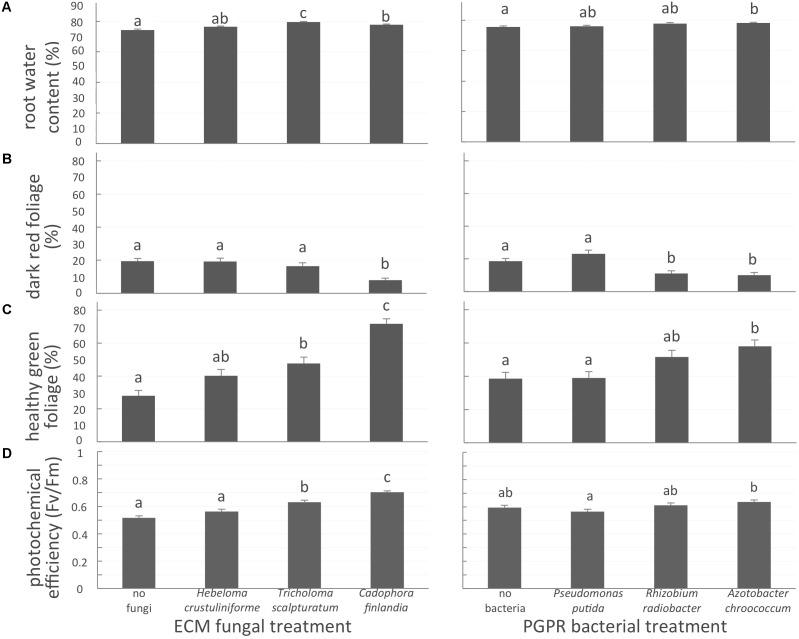
White spruce health related parameters affected by symbiotic associations. **(A)** Root water content (%); **(B)** proportion of dark-red foliage (%); **(C)** proportion of healthy green foliage (%), and **(D)** photochemical efficiency (Fv/Fm ratio) were affected by both mycorrhizal fungal treatment and bacterial treatment after 32 weeks of growth. Values are means ± SE. Different letters indicate significant difference.

Belowground, the inoculation of seedlings with one of the two native mycorrhizal fungi *T*. *scalpturatum* and *C. finlandia*, increased root water content by 6 and 4%, respectively, when compared to non-inoculated control plants (**Figure 2A**, left panel; *P*-values < 0.0001). Despite a 2% increase, seedlings inoculated with *H. crustuliniforme* did not differ statistically from control. For the bacterial treatments, only *A*. *chroococcum*-associated seedlings outperformed non-inoculated control plants (**Figure 2A**, right panel; *P*-values = 0.003).

The proportion of dark-red foliage is a health-related variable indicative of element toxicity; a higher percentage value indicates reduced seedling health. In the control seedlings, almost one-third (31 ± 2%; *P*-values < 0.0001) of seedling foliage was dark red. This suggests the importance of the symbiotic associations with either fungal partner or a bacteria strain for plant health in a severely disturbed environment. Indeed, plants inoculated with *C*. *finlandia* exhibited significantly less dark-red foliage than controls (**Figure 2B**, left panel; *P*-value < 0.0001). The proportion of healthy green foliage on seedlings inoculated with *C*. *finlandia* and *T. scalpturatum* was significantly greater than control plants without fungal inoculation (**Figure 2C**, left panel; *P*-value < 0.0001). The benefit of the allochthonous fungus *H*. *crustuliniforme* was not significant, a trend we also observed for photochemical efficiency (**Figure 2D**, left panel).

For bacterial treatments (**Figures 2C,D**, right panels; *P*-values < 0.0001), only the *A. chroococcum* commercial strain significantly increased the proportion of healthy-green foliage and to a slightly lower extent, association with the locally sourced-bacteria *R. radiobacter* compared to control and *P. putida* treatments (**Figure 2B**, right panel; *P*-value < 0.0001).

Photochemical efficiency (Fv/Fm) measures the capacity of the photosystem apparatus to capture light energy. From 0.56 in control plants, Fv/Fm ratio did not improve in *H*. *crustuliniforme*-associated plants (0.58) but reached 0.63 and 0.70 in plants associated with *T*. *scalpturatum* and *C*. *finlandia* (*P*-value < 0.0001). Association with the *A*. *chroococcum* commercial strain also improved photochemical efficiency compared to *P*. *putida*-associated plants (*P*-value = 0.0048). In fact, *P*. *putida* did not improve any of the four health-related parameters measured.

### Effect of Symbiotic Association on Plant Growth

**FIGURE 3:**
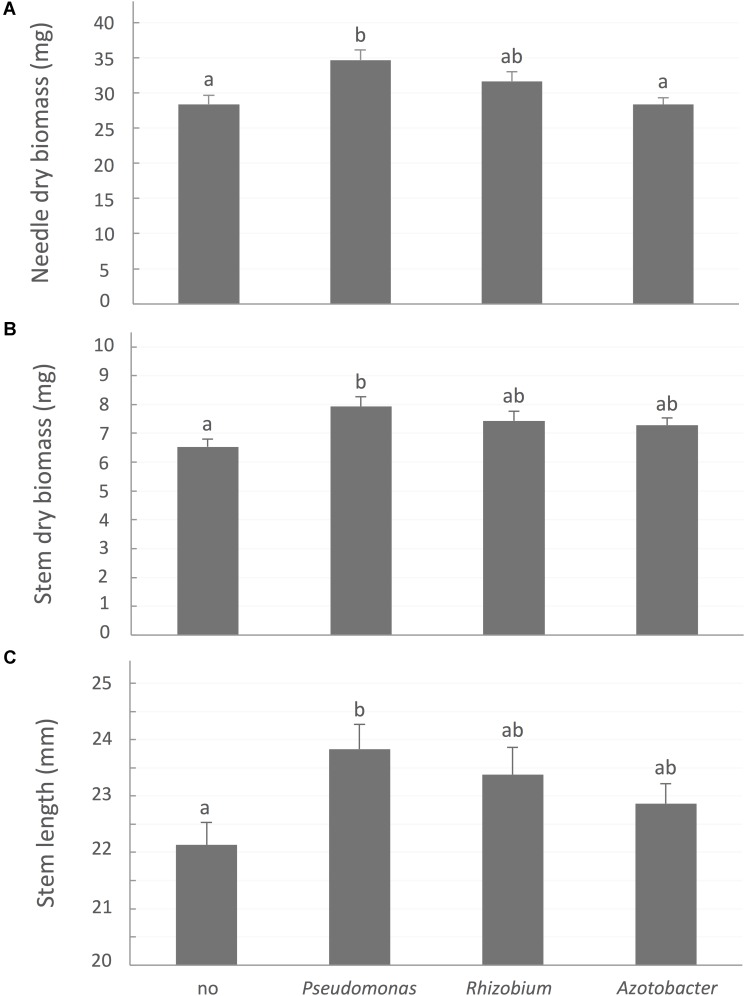
White spruce growth parameters affected by association with bacteria. **(A)** Needle dry biomass (mg), **(B)** Stem dry biomass (mg), and **(C)** stem length (mm) were affected by rhizobacterial treatments after 32 weeks of growth. Values are means ± SE. Different letters indicate significant difference.

*A contrario*, bacterial treatments, especially site-specific species, significantly improved aboveground growth (**Figure 3**). After 32 weeks, seedlings inoculated with *P*. *putida* had significantly greater needle biomass (*P*-value < 0.0001), stem biomass (*P*-value = 0.0048), and stem length (*P*-value = 0.0217) than control plants not associated with a bacterial strain.

**FIGURE 4:**
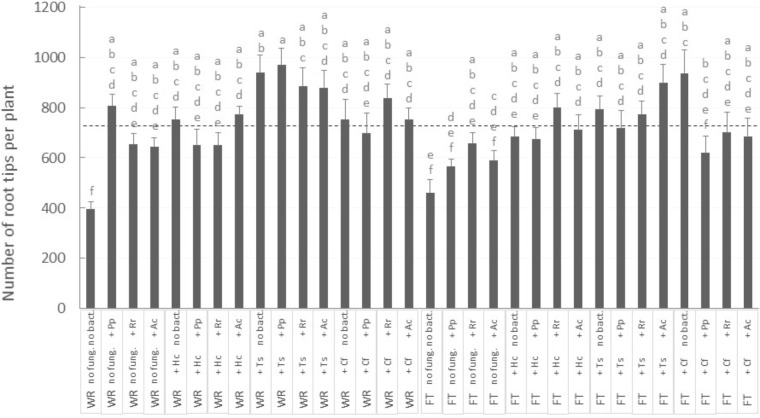
Number of white spruce root tips after 32 weeks of growth in 32 different conditions. There was an interaction between the three factors (*P-*value = 0.0128): tailing type (WR, waste rocks; FT, fine tailings); mycorrhizal fungi (no fung., no fungi; +Hc, *Hebeloma crustuliniforme*; +Ts, *Tricholoma scalpturatum*; +Cf, *Cadophora finlandia*); and bacteria (no bact., no bacteria; +Pp, *Pseudomonas putida*; +Rr, *Rhizobium radiobacter*; +Ac, *Azotobacter chroococum*). The two large light gray bars indicate average root tips number on waste rocks and FTs. Values are means ± SE, same letters are not significantly different at α = 0.05, Tukey test.

Surprisingly, no aerial growth parameter was directly influenced by mycorrhizal fungi treatment. However, careful observation of the belowground growth was much more informative. For the number of root tips per plant, there was an interaction between the three factors *Tailing type* × *fungus* × *bacteria* (**Figure 4**; *P*-value = 0.0128), while root tips number was <400 in plants grown on WR and without microorganism associations: this number increased significantly by 60 to 140% (**Figure 4**, “WR” labeled bars on the left) with the inoculation of at least one symbiont (fungus, bacteria, or both). The association with native fungi were the most beneficial: on WRs, number of root tips went consistently above average (**Figure 4**, see dashed line) when plants were associated with *T*. *scalpturatum*. On FTs (**Figure 4**, “FT” labeled bars on the right), the number of root tips increased by 20 to 100% (from 461 when plants were grown without a partner) but only when seedlings grew in association with some specific symbiont combinations. On this tailing type, the association with *T. scalpturatum* was also beneficial but the inoculation of rhizobacteria was not sufficient to notice a significant increase; neither did the allochthonous fungus *H. crustuliniforme* alone and several other fungus × bacteria combinations.

### Mycorrhizal Root Colonization Rate

**FIGURE 5:**
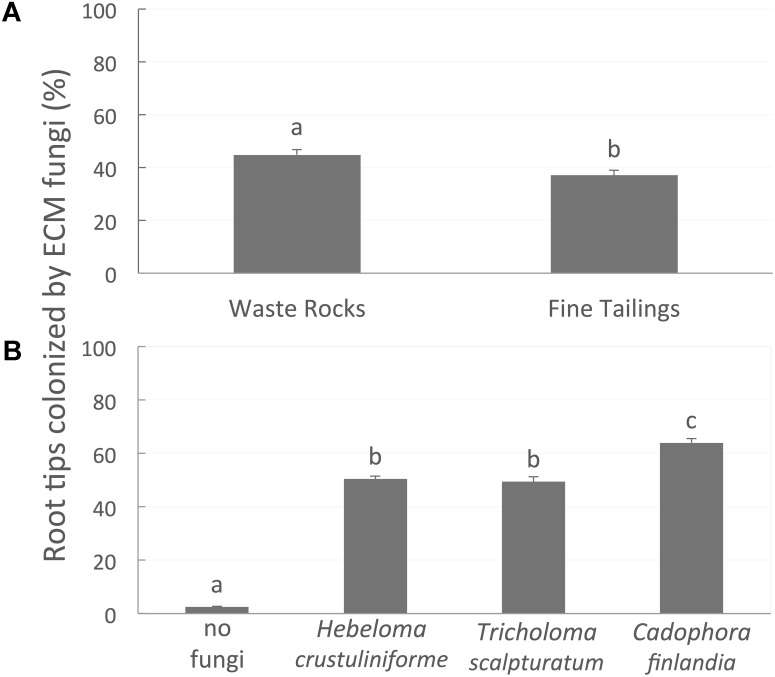
The proportion of root tips colonized by mycorrhizal fungi. **(A)** Figure is different on waste rocks than on fine tailings, **(B)** diverge between the different fungi inoculated. Values are means ± SE. Different letters indicate significant difference.

Root colonization was greater on WRs (45%) than on FTs (37%) (**Figure 5A**; *P*-value < 0.0001). The percentage of root tips colonized was not influenced by bacterial treatment (*P*-value = 0.3194) but varied between the different mycorrhizal fungi (**Figure 5B**; *P*-value < 0.0001). For instance, seedlings associated with *C*. *finlandia* displayed the greatest percentage of colonized root tips (63%).

Interestingly, the proportion of root tips colonized correlated with health and growth variables in this experiment (**Table 1**). The proportion of healthy green foliage, photochemical efficiency, root growth parameters (total biomass, volume, stem length, and root tips number), stem and finally needle dry biomass correlated positively with the percentage of root tips colonized. However, those relationships broke for several growth parameters (number of root tips, stem, or needle dry biomass) when plants were associated with the allochthonous *H. crustuliniforme* and tended to be significantly much stronger with *C. finlandia* than with *T. scalpturatum*.

**Table 1:** Pearson correlation coefficient (*r*) and their associated *P-*values calculated for the proportion of root tips colonized by different mycorrhizal fungi and measured health and growth variables.

	Proportion of healthy green foliage			Photochemical efficiency		
*Cadophora finlandia*	***r* = 0.75** (*P*-value < 0.0001)			***r* = 0.67** (*P*-value < 0.0001)		
*Tricholoma scalpturatum*	***r* = 0.71** (*P*-value < 0.0001)			***r* = 0.54** (*P*-value < 0.0001)		
*Hebeloma crustuliniforme*	***r* = 0.47** (*P*-value < 0.0001)			***r* = 0.33** (*P*-value = 0.0014)		

	**Root dry biomass**	**Total root volume**	**Total root length**	**Root tips number**	**Stem dry biomass**	**Needle dry biomass**

*Cadophora finlandia*	***r* = 0.56** (*P*-value < 0.0001)	***r* = 0.59** (*P*-value < 0.0001)	***r* = 0.49** (*P*-value < 0.0001)	***r* = 0.34** (*P*-value = 0.0013)	***r* = 0.38** (*P*-value = 0.0004)	***r* = 0.34** (*P*-value = 0.0014)
*Tricholoma scalpturatum*	***r* = 0.43** (*P*-value < 0.0001)	***r* = 0.47** (*P*-value < 0.0001)	***r* = 0.41** (*P*-value < 0.0001)	***r* = 0.26** (*P*-value = 0.015)	***r* = 0.28** (*P*-value = 0.0078)	***r* = 0.21** (*P*-value = 0.0451)
*Hebeloma crustuliniforme*	***r* = 0.37** (*P*-value = 0.0003)	***r* = 0.41** (*P*-value < 0.0001)	***r* = 0.35** (*P*-value = 0.0008)	*r* = 0.15 (*P*-value = 0.1714)	*r* = 0.2 (*P*-value = 0.0606)	*r* = 0.12 (*P*-value = 0.2653)

*Significant correlation at α ≤ 0.05. Statistically significant values (P < 0.05) are given in bold.*

## Discussion

In the present study, tree-symbiont associations proved to be beneficial to the establishment of white spruce seedlings on waste material of the Sigma-Lamaque gold mine. Mine-adapted mycorrhizae and rhizobacteria were respectively capable of improving plant health and growth under glasshouse conditions.

### Microbial Symbiotic Association Improved Overall Plant Health

*Azotobacter chroococcum* and to a lesser extent *R*. *radiobacter* were more successful in enhancing white spruce seedlings health but much less than mycorrhizal fungi and *C*. *finlandia* in particular. Indeed, seedling health was greatly improved by the two fungi *C. finlandia* and *T. scalpturatum* isolated from the mining site. Inoculation with *H*. *crustuliniforme* neither improved root water uptake nor other plant health parameters, albeit Onwuchekwa et al. (2014) have demonstrated that *H*. *crustuliniforme* increased white spruce water uptake on oil sand tailings. Moreover, the same strain we used in this study had proven to be beneficial to seedlings grown on peat moss and sand mix under salt stress conditions (Mushin and Zwiazek, 2002). But as a drought intolerant strain isolated from a natural boreal forest stand (Coleman and Bledsoe, 1989), *H*. *crustuliniforme* may not be well-suited to grow in mine tailings, an environment very prone to water stress. Native *C*. *finlandia* and *T*. *scalpturatum* fungi may be better adapted, thereby capable of enhancing seedling health. Both species are commonly found in high abundance and frequency, in heavy metal polluted sites (Krpata et al., 2008; Gorfer et al., 2009). Extreme, arid, or toxic soil conditions lead to the evolution of tolerant strains, an adaptation indispensable for both tree and fungal survival (Colpaert et al., 2011). Like for many fungi (Douhan et al., 2011), the genetic diversity of *C*. *finlandia* is still unknown but, under conditions of high heavy metal concentrations, it has the capability to enhance the expression of several genes encoding extracellular and plasma membrane proteins potentially involved in detoxification processes (Gorfer et al., 2009). *T*. *scalpturatum* is a generalist species with high genetic diversity at the local scale (Carriconde et al., 2008), whereas, *H. crustuliniforme* has low intraspecific genetic diversity (Aanen et al., 2000).

The ability of mycorrhizae to improve seedling health is indeed site-specific and positively associated with its capacity to grow on a given substrate. Root colonization rate was much lower on FTs than on WRs, the substrate on which plants performed the best. In a preliminary *in vitro* experiment (unpublished), we have found that *C*. *finlandia* produced the highest mycelial biomass on poor liquid medium amended with mine tailings followed by *T*. *scalpturatum* and then *H*. *crustuliniforme*. Accordingly, in the present study, plants inoculated with *H*. *crustuliniforme* were the ones with the least colonized root system compared to *T*. *scalpturatum* and *C*. *finlandia* inoculated ones, the later being the most colonized. *C*. *finlandia* is also the one symbiont that alleviated the most Ca and Fe deleterious effects *in vivo* (Nadeau et al., 2018). Health-related parameters were strongly and positively correlated with root colonization rates of the two fungi isolated from the mining site but only to a smaller extent with mycorrhization rate of *H*. *crustuliniforme*, the fungus that presents the lowest potential for reforestation program in gold mine WR and FTs.

### Improvement of Plant Growth Is Associated With Symbiont Type and Source

Despite its huge effect on plant health, mycorrhization did not enhance seedling aerial growth in this experiment. Moreover, mycorrhization rate of native species correlated well with root growth but much less with aerial growth parameters. Early root colonization could have a carbon cost that negates the aboveground seedling growth during the first growing season but enhances it the subsequent years (Rygiewicz and Andersen, 1994). An idea supported by the fact that several treatments with at least one symbiont yielded a higher number of fine root tips on white spruce seedlings than the control without symbiont. Mycorrhizal fungi and rhizobacteria such as species of the genus *Pseudomonas* and *Azotobacter* produce auxins that alter considerably the host root morphology (Rajkumar et al., 2009; Etemadi et al., 2014). On that account, hormone production by the investigated symbionts plays probably a very important role in plant growth behavior. The production of a higher number of root tips by seedlings inoculated with mycorrhizal fungi and/or rhizobacteria may be highly beneficial to white spruce trees by allowing extra uptake of water and nutrients.

The inoculation of white spruce with *P*. *putida*, a rhizobacteria strain isolated from the rhizosphere of healthy white spruce seedlings naturally regenerating on the mining site, was the only treatment that increased considerably seedling aerial growth. Beall and Tipping (1989) and O’Neill et al. (1992) have also reported that *P*. *putida* enhanced jack pine and spruce aerial growth.

Though the beneficial effects of *A*. *chroococcum* or *R*. *radiobacter* have been extensively demonstrated in both woody (Leyval and Berthelin, 1993; Karthikeyan and Sakthivel, 2011) and non-woody (Aquilanti et al., 2004; Humphry et al., 2007; Baset Mia and Shamsuddin, 2010) plant species, neither of the commercial strain of *A. chroococcum* nor the indigenous *R*. *radiobacter* influenced plant growth according to our results. However, to the best of our knowledge, the present study is the first to investigate the impact of native rhizobacteria on gold mine tailings, a completely different environment than agricultural fields and forest stands. Indeed *A*. *chroococcum* may not be adapted to tailing conditions as much as *P*. *putida* in that perspective. However, unlike *P*. *putida*, both *A*. *chroococcum* and *R*. *radiobacter* had appreciable effects on seedling health suggesting a much complex explanation for their limited plant growth promoting ability. For that reason, bacterial selection must be cautiously done in order to identify strains that have the potential to be effective in the field.

### Selection of Symbiotic Partners for Successful Mine Reforestation Programs

In conclusion, our initial on-site sampling strategy proves to be effective: mycorrhizal fungi and rhizobacteria isolated from roots of healthy white spruce seedlings naturally regenerating on the mining site proved to be remarkably more efficient than allochthonous species in enhancing seedling health and growth when planted on mine tailings. Within the 32 weeks of glasshouse trial, native fungi, *C*. *finlandia*, *T*. *scalpturatum*, and the native rhizobacteria *R*. *radiobacter* promoted seedling health through better root colonization rate. The native rhizobacteria *P. putida* was the sole symbiont that distinctly improve seedling aerial growth but not seedling health. Allochthonous fungus, *H. crustuliniforme* had little effects on seedling performance, whereas plants benefited from the inoculation of *A. chroococcum*. Specifically, the allochthonous rhizobacteria *A. chroococcum* improved seedling root water uptake, especially when paired with mycorrhizal fungi. As discussed in a companion paper (Nadeau et al., 2018), a symbiont (or symbiont combination) capacity to modulate plants access to otherwise limited (water, nutrients) or toxic compounds is directly linked to the success of the white spruce seedling establishment.

Since its soil chemical composition is already well-documented, the Sigma-Lamaque gold mine at Val-d’Or is at one and the same time a land that must be revegetated once its exploitation is completed and a potential testing ground to validate the several steps involved in the development of a new green technology (Nadeau, 2014). The comprehensive analysis of the entire ecosystem – of which the present study is an important component – should unravel the significance of each parameter for the success of an integrated reforestation program including the soil chemical composition and the isolation, selection, validation, and large-scale production of the best plant–symbiont combinations (Nadeau and Khasa, 2016).

## Author Contributions

MBN and DK conceived and designed the experiments. MBN performed the experiments. MBN, JL, and DK analyzed the data and wrote the manuscript.

## Conflict of Interest Statement

The authors declare that the research was conducted in the absence of any commercial or financial relationships that could be construed as a potential conflict of interest.

## Footnotes

1 http://www.cef-cfr.ca/index.php?n=CEF.Collections

## Supplementary Material

FIGURE S1 Illustrations and design of the experiment. Mycorrhizal fungi and rhizobacteria used in this **(A)** glasshouse experiment were either **(B)** native from the Sigma-Lamaque gold mine site [*Tricholoma scalpturatum* (Ts), *Cadophora finlandia* (Cf), *Pseudomonas putida* (Pp), *Rhizobium radiobacter* (Rr)] or isolated from a natural forest stand [*Hebeloma crustuliniforme* (Hc)] or of commercial origin for *Azotobacter chroococcum* (Ac). White spruce saplings were either planted in **(C)** waste rocks (WRs) or in fine tailings (FTs). **(D)** Randomized complete block (RCB) design with three crossed fixed factors: tailing type (FT in regular font, WR in bold); fungi [none (noF) in black; Ts, green font; Cf, blue font; Hc, red font]; and bacteria [none (noB), no background; Rr, background in red; Pp, background in blue; Ac, background in green] for a total of 32 treatments, 4 blocks, 3 replicates per treatment per block, and 384 experimental units.
