# Supplementary figures and images for: Mycorrhizae and Rhizobacteria on Precambrian Rocky Gold Mine Tailings: I. Mine-Adapted Symbionts Promote White Spruce Health and Growth
Source: Front Plant Sci. 2018 Sep 3;9:1267. doi: 10.3389/fpls.2018.01267 (PMC6130231; doi:10.3389/fpls.2018.01267)

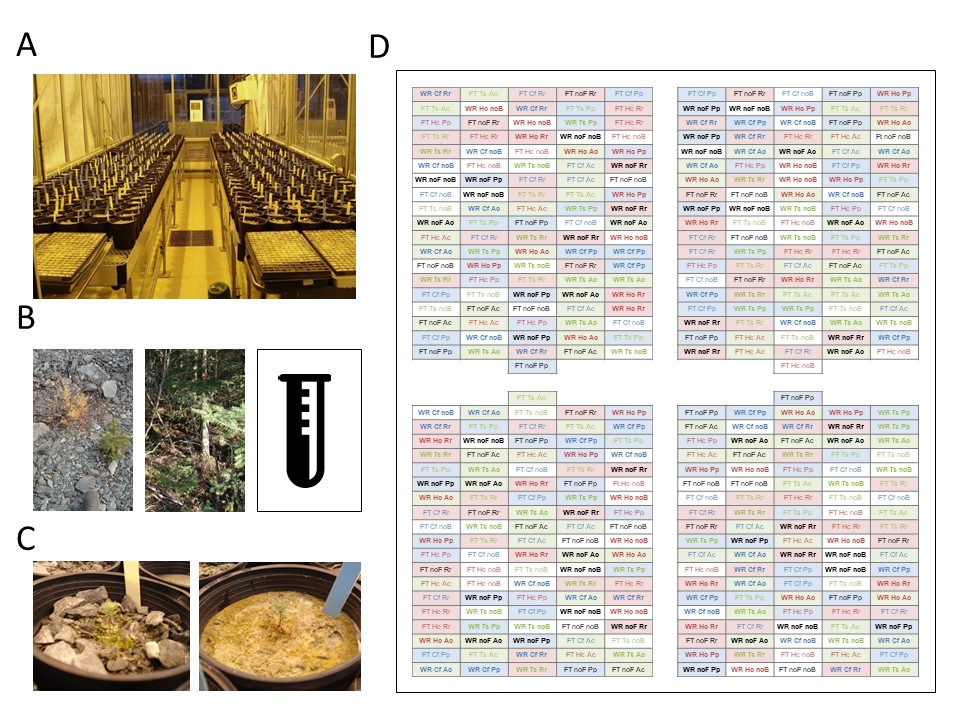

Supplement: FIGURE S1 — Illustrations and design of the experiment. Mycorrhizal fungi and rhizobacteria used in this (A) glasshouse experiment were either (B) native from the Sigma-Lamaque gold mine site [Tricholoma scalpturatum (Ts), Cadophora finlandia (Cf), Pseudomonas putida (Pp), Rhizobium radiobacter (Rr)] or isolated from a natural forest stand [Hebeloma crustuliniforme (Hc)] or of commercial origin for Azotobacter chroococcum (Ac). White spruce saplings were either planted in (C) waste rocks (WRs) or in fine tailings (FTs). (D) Randomized complete block (RCB) design with three crossed fixed factors: tailing type (FT in regular font, WR in bold); fungi [none (noF) in black; Ts, green font; Cf, blue font; Hc, red font]; and bacteria [none (noB), no background; Rr, background in red; Pp, background in blue; Ac, background in green] for a total of 32 treatments, 4 blocks, 3 replicates per treatment per block, and 384 experimental units. [file Image_1.JPEG]
